# Assessing the inhibitory activity of culture supernatants against foodborne pathogens of two psychrotrophic bacteria isolated from river trout

**DOI:** 10.1007/s00203-022-02919-5

**Published:** 2022-05-04

**Authors:** Carla Condò, Irene Gómez, Maribel Farfán, Núria Rius

**Affiliations:** 1 Department of Biology, Healthcare and the Environment, Faculty of Pharmacy and Food Sciences, University of Barcelona, Av. Joan XXIII 27-31, 08028 Barcelona, Spain; 2 The Biodiversity Research Institute (IRBio), University of Barcelona, Av. Diagonal 645, 08028 Barcelona, Spain; 3 Institute for Nutrition and Food Safety (INSA-UB), University of Barcelona, Av. Prat de la Riba 171, 08921 Santa Coloma de Gramenet, Spain

**Keywords:** Antimicrobial activity, EPS production, Food preservation, *Pseudomonas fragi*, *Shewanella baltica*

## Abstract

There is a need for new natural products with antimicrobial activity to treat multidrug resistant bacteria that can cause human illness. Some of them are foodborne pathogens. Two different Gram-negative psychrotrophic strains were isolated from healthy trout river samples (*Salmo* *trutta*). Based on phenotypic characterization, proteomics, genotyping and phylogenetic analyses of 16 rRNA gene, strains TCPS12 and TCPS13 were identified as *Shewanella* *baltica* and *Pseudomonas* *fragi*, respectively. Both of them produced an exopolysaccharide that showed antimicrobial activity against four foodborne pathogens. *P. fragi* supernatant (AS13) showed higher antimicrobial activity than *S. baltica* supernatant (AS12) against all tested pathogens. The stability of the antimicrobial activity of AS13 was assessed against *Enterococcus faecalis* ATCC 29212 under different conditions. This solution was stable when exposed for 30 min to temperatures ranging from 40 to 100 °C. In addition, it retained its activity within a pH range of 2–8 during 2 h of incubation, showing higher activity at pH 6. Serine proteases and α-amylase inactivated significantly the antimicrobial activity of AS13, suggesting that the active molecule could most likely be a glycoprotein. These products are interesting for their possible application as biopreservatives in the food industry.

**Supplementary Information:**

The online version contains supplementary material available at 10.1007/s00203-022-02919-5.

## Introduction

Antibiotics have been used both to prevent and treat microbial diseases. In the intensive animal farms needed for food production, they are used not only to treat animal diseases but also to prevent infections and to increase animal’s growth rates (Kirchhelle 2018; Smith 2015; Stoica and Cox 2021; Van Boeckel et al. 2015). It has been demonstrated that the abuse of antimicrobials increases the number of drug-resistant bacteria. The number of bacteria acquiring multidrug resistance increases every day with it now being one of the biggest issues of human health, food safety and socioeconomic development (Smith De Waal et al. 2011; Wenzel 2004). Soil and water are contaminated by antimicrobial residues contained in animal wastes, increasing the risk for dissemination of multidrug-resistant microorganisms (Cycon et al. 2019; Food and Agriculture Organization of the United Nations 2017; He et al. 2020). Treatment of diseases caused by multidrug resistant bacteria is often difficult or even impossible. This leads to an increment of both morbidity and mortality rates, also in developed countries (Mancilla-Becerra et al. 2019).

Most foods, both from plant and animal origin, can be contaminated with antibiotic-resistant microorganisms thorough the food chain, from the farm to the consumer. Some of them are human pathogens that can cause foodborne illness (Lucera et al. 2012; Mancilla-Becerra et al. 2019). In addition, the genes encoding for the resistance can be transferred to other bacteria increasing exponentially the number of multiresistant bacteria. This represents one of the biggest issues for human health and global economy (Mancilla-Becerra et al. 2019; Projan 2003; Smith De Waal et al. 2011; Wenzel 2004) so the discovery of new products coming from natural sources could be useful to deal with this tough problem.

Microorganisms isolated from aquatic systems, both freshwater and fish, tolerate extreme environmental conditions. Most of them possess genetic elements that have only been detected in these kind of habitats (Jin et al. 2019). There are bacteria and fungi able to produce secondary metabolites, i.e. antimicrobial compounds, that could be of interest to the food industry (Li et al. 2014; Naghmouchi et al. 2011).

The aims of our work were to isolate psychrotrophic bacteria from healthy river trout samples, characterize and identify them by both classical and molecular methods, and to find out the production of an exopolysaccharide (EPS) with antimicrobial activity against foodborne pathogens.

## Materials and methods

### Reagents and media

All chemical reagents were from Panreac Quimica SLU (Castellar del Vallès, Spain). Ringer solution, Buffered Peptone Water (BP), Tryptone Soy Broth (TSB), Nutrient Broth (NB), Mueller Hinton Broth (MH) and Pseudomonas cetrimide agar were from Oxoid LTD (Bashingstoke, UK). Trypticasein Soy Agar (TSA) was from Pronadisa (Laboratorios Conde S.A., Madrid, Spain). SIM medium was from Difco (Fisher Scientific S.L., Madrid, Spain). King A agar and King B agar were from Scharlau Chemie S.A. (Barcelona, Spain). Columbia agar supplemented with 5% of sheep blood was from Oxoid (Thermo Fisher Diagnostics S.L.U., Madrid, Spain). Phosphate Buffered saline (PBS) was prepared as follows: NaCl (8 g L^−1^), KCl (0.2 g L^−1^), Na_2_HPO_4_ (1.44 g L^−1^), KH_2_PO_4_ (0.24 g L^−1^). α-Amylase from *Bacillus licheniformis*, lipase from porcine pancreas, proteinase from *Tritirachium album*, α-chymotrypsin from porcine pancreas and trypsin from bovine pancreas were from Sigma (Sigma-Aldrich, St. Louis, MO, USA).

### Microbial strains and culture conditions

*Enterococcus faecalis* ATCC 29212, *Staphylococcus* *aureus* ATCC 9144, *Escherichia coli* ATCC 25922 and *Salmonella* Enteritidis ATCC 49214 were used to test the antimicrobial activity of our isolates. *Pseudomonas* *aeruginosa* ATCC 27853 and *Pseudomonas* *fluorescens* ATCC 13430 were used as control strains in *Pseudomonas* biochemical tests. All strains were obtained from CECT (Colección Española de Cultivos Tipo). They were grown on TSA incubated 24 h at 30 °C, stored at 4 °C, and streaked on the same medium every 2 weeks.

### Isolation of microorganisms from fish samples

Samples of river trout (*Salmo* *trutta*) were collected from the local market La Boqueria (Barcelona, Spain) and stored for a maximum of 2 h at 4 °C. A portion of 30 g of 127.3 g fish samples including meat and skin but not guts were placed in a mortar with 300 mL of buffered peptone water and amalgamated with a pestle. Mortar and pestle were previously sterilized with ethanol absolute. The obtained suspension was collected in sterile bottles and serially twofold diluted in sterile Ringer ¼ solution. 100 µL of each dilution were spread on TSA plates and incubated for 24 h at 30 °C. After incubation, several microbial colonies that showed mucus morphology were streaked on TSA plates to obtain pure cultures. The isolated microorganisms from fish samples were stored in sterile Eppendorf tubes at − 80 °C in TSB containing 20% glycerol until required. Strains TCPS12 and TCPS13 have been deposited in the Spanish Type Culture Collection (CECT).

### Phenotypic characterization

One Eppendorf tube of each strain was defrosted at room temperature, streaked on TSA plates and incubated for 24 h at 30 °C. One colony of each strain was cultured in Nutrient Broth and incubated 24 h at 30 °C. Cultures were then centrifuged 5 min at 7686*g* at room temperature (Centrifuge 5415D, Eppendorf AG 22331 Hamburg). Pellets were washed three times with Ringer ¼ sterile solution and suspended in the same solution. The obtained suspensions were used for the determination of both microbial morphology and biochemical characterization.

Bacteria morphology was determined by gram-staining and confirmed by non-staining KOH method (Buck 1982). Oxidase test, motility and H_2_S production in SIM agar at 20 °C and 30 °C, nitrate reduction, hemolysis on Blood agar, and growth in anaerobic conditions at 30 °C for 14 days on TSA plates using GasPak System (Oxoid, Bashingstoke, UK) were used to characterize both strains. Physiological characterization and carbon source utilization were determined using API 20E and API 20NE identification Systems (AES CHEMUNEX S.A., Terrassa, Spain). Production of pyocyanin and pyoverdine was studied on Cetrimide plates, King A agar and King B agar slants. All biochemical tests were conducted at 30 °C. *P. aeruginosa* ATCC 27853 and *P. fluorescens* ATCC 13430 were used as control strains.

Aerobically growth of the isolates at 4 °C, 15 °C, 20 °C, 30 °C, 35 °C and 37 °C was tested on TSB and TSA. Buffered peptone water with 0%, 1%, 3%, 5%, 8%, 10%, 12% and 15% NaCl was used to test the tolerance of the isolates to NaCl. The pH of these media was controlled before use (Crison, micropH2001, HACH LANGE SPAIN S.L.U. L’Hospitalet de Llobregat, Spain). Cultures were incubated at 20 °C.

### MALDI-TOF MS and phylogenetic analyses

Bacterial identification by MALDI-TOF mass spectrometry and DNA sequencing of 16S rRNA were performed by Aconsa^®^ company (Asesoría y Consultoría S.L., Sant Joan Despí, Barcelona, Spain) from pure cultures of the selected strains. Sequence data were queried against archived sequences in the GenBank database with the BLASTN program (http://www.ncbi.nlm.nih.gov). The 16S rRNA gene sequences obtained in this study have been deposited in the GenBank database under accession numbers MN822711 (strain TCPS12) and MN822712 (strain TCPS13).

Multiple sequence alignments were performed using the ClustalW program implemented in the MEGA7 software package (Kumar et al. 2016). The 16S rDNA nucleotide sequences of both isolates were compared with those sequences of closely related species available in the GenBank. The model of evolution was determined with MEGA7, using the Kimura-2-parameter (K2) model as the best-fit model of nucleotide substitution for each dataset. Neighbor-joining phylogenetic trees were reconstructed using MEGA7 software with 1000 bootstrap replicates to assess tree topology robustness.

### Preparation of cell-free culture supernatants

An inoculum of each isolate was prepared in Ringer ¼ solution with a preculture on TSA at 30 °C. The optical density (O.D.) of each suspension at 580 nm was adjusted at 2 using a Shimadzu spectrophotometer UV-1800 (Shimadzu Corporation, Kyoto, Japan). 500 mL baffled Erlenmeyer flasks were filled with 100 mL of the growing mineral salts medium as described by Burgos-Díaz et al. (2011) with 2.5% glucose as carbon source and 2% (v/v) of the inoculum. Cultures were incubated aerobically 72 h at 30 °C and 120 rpm in an orbital shaker. After the incubation period each microbial culture was centrifuged at 98.03*g* for 15 min at 5 °C in an Allegra^™^ 5R centrifuge (Rotor A-10, Beckman Coulter™, USA). Supernatants (AS) were freeze-dried (Cryodos^™^ 50, Telstar, Terrassa, Spain). Each 20 mL of lyophilized supernatants were resuspended in 2 mL of sterile PBS before use. Supernatants obtained from TCPS12 and TCPS13 strains were named AS12 and AS13, respectively.

### Antimicrobial activity of supernatants (AS)

The antimicrobial activity of both AS12 and AS13 was evaluated by the microdilution method in sterile 96 well polystyrene plates. In each plate, three wells were filled with 20 µL of AS and 180 µL of the four test strains in MH broth (5 × 10^4^ cfu per well, final concentration). 180 µL of sterile MH broth were added to the first well together with 20 µL of each AS (sterility controls). Positive controls consisted on 20 µL of sterile PBS plus 180 µL of test cultures in MH broth. The pH of experimental wells was 6. To study the influence of pH on the growth of test strains, three wells containing each 20 µL of sterile PBS at both pH 7 and at pH 3 and 180 µL of each microbial culture in MH broth were prepared. Each microplate was thus prepared in duplicate. The microplates were incubated 24 h at 37 °C. After incubation, wells were thoroughly mixed, and O.D. at 580 nm was determined on a Shimazdu UV Spectrophotometer (Shimazdu Corporation, Kyoto, Japan) with an automated Synergy microplate Reader (Biotek Instruments Inc., Winooski, VT, USA). The inhibitory activity (I. A.) was calculated as percentage according to the indications reported by Naghmouchi et al. (2011) using the following formula:

$${\text{I}}.{\text{A}}.\, = \,{\text{1}}00{-}{\text{1}}00{\text{ }}[{\text{OD}}_{{{\text{58}}0}} \left( {\text{s}} \right)/{\text{OD}}_{{{\text{58}}0}} \left( {{\text{ns}}} \right)],$$

where (s) is the culture containing the AS and (ns) is the positive control culture. The results were reported as means of two independent analyses.

### Stability of the antimicrobial solution AS13

The stability of the AS13 antimicrobial activity at different temperatures and pH values was evaluated against *E. faecalis* ATCC 29212. Thermal stability was determined by exposing AS to temperatures ranging from 40 to 100 °C, at intervals of 10 °C, in a water bath for 30 min. To test the effect of pH on antimicrobial activity of supernatants, samples of AS were adjusted to pH 2, 4, 6, 8, and 10 with 0.1 N HCl or 0.1 N NaOH, and incubated at 25 °C for 2 h. The samples were neutralized to the original pH before testing for antimicrobial activity (Alkotaini et al. 2014). The effect of temperature and pH on the antimicrobial activity was studied by the microdilution method in duplicate as explained before. The I.A. of the treated supernatants against *E. faecalis* ATCC 29,212 was calculated in comparison with untreated supernatants. Control conditions of AS (100% of antimicrobial activity) were 6.0 pH units and 25 °C. All analyses were performed twice.

### Effect of proteolytic enzymes on antimicrobial activity of AS13

Antimicrobial activity of AS13 was tested after treating AS 2 h at 37 °C with 2.5 mg mL^−1^ (final concentration) of α-chymotrypsin (Q), lipase (L), trypsin (T), amylase (A), or 1 mg mL^−1^ (final concentration) of proteinase K (P) according to Naghmouchi et al. (2011) by the microdilution method. All treatments were performed in duplicate with three different supernatants except for the treatment with amylase, which was performed with two supernatants.

### Statistical analysis

A multifactor analysis of variance (ANOVA) was used to test for significant differences between supernatants activity, pH and enzyme treatments (*p* < 0.05).

## Results and discussion

### Phenotypic characterization

Fifteen strains producing an exopolysaccharide (EPS) on TSA plates with antimicrobial activity against foodborne pathogens were isolated from river trout samples. Among them, two strains showing different colony morphology exhibited more antibacterial activity against both Gram-negative and Gram-positive pathogens. As shown in Supplementary Table S1, both strains were Gram-negative rods, oxidase positive, non-hemolytic, motile, psychrotrophic (with a grow temperature range from 4 to 35 °C, but unable to grow at 37 °C), and showed no growth in anaerobic conditions. TCPS12, characterized by a yellow color of its colonies on TSA plates, was able to produce H_2_S, showed nitrate reduction activity and a growth in the presence of NaCl ranging from 0 to 8%, but not with 10% NaCl. TCPS13, characterized by a white color of its colonies on TSA plates, did not show H_2_S production, could not reduce nitrate, was able to grow without NaCl to 5% NaCl, but not with 8% NaCl and did not show pyocianin and pyoverdine production. The TCPS12 strain was presumptively identified as *Shewanella putrefaciens* in the API 20E database, while the TCPS13 strain was presumptively identified as *Pseudomonas* spp. in the API 20E database and as *Pseudomonas putida* in the API 20NE database. Our results agree well with those reported by Gram and Huss (1996), Skrodenyte-Arbaciauskiene et al. (2006) and Venugopal et al. (1990).

### MALDI-TOF MS and phylogenetic analyses

A polyphasic approach was conducted, including proteomics, genotyping and phylogenetic analyses, to clarify the taxonomic position of both isolates at species level. The combination of MALDI-TOF MS and 16S rDNA sequencing was used to allow the identification of bacterial species with higher accuracy (Gomila et al. 2015; Holt et al. 2005; Owen et al. 1978; Palleroni 2015; Ziemke et al. 1998).

**Fig. 1:**
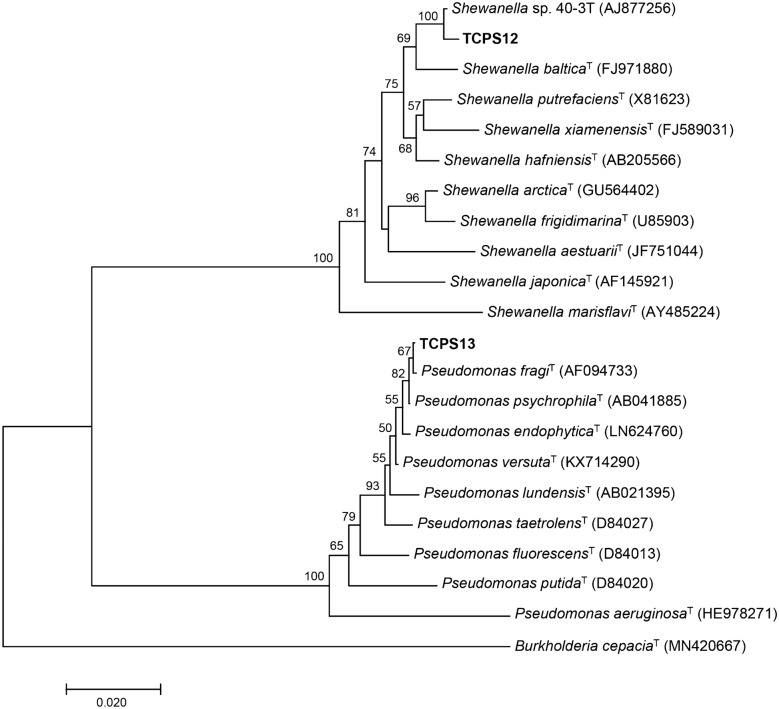
Neighbor-joining phylogenetic tree based on 16S rRNA gene sequences, showing the relationships between the strains TCPS12 and TCPS13 and the type strains of closely related species of the genera *Shewanella* and *Pseudomonas,* respectively. The strain 40-3 T has been included as the most related member to TCPS12. *Burkholderia cepacia* was used as out-group. Bootstrap values higher than 50% are indicated on the corresponding nodes. Scale bar represents the number of substitutions per nucleotide position

The almost complete 16S rRNA sequences of the two studied strains TCPS12 (1144 bp) and TCPS13 (1136 bp) were obtained and compared with published sequences from the database using BLASTN analysis. Sequence similarity values obtained were above the cut-off value of 98.7% for delineating bacterial species (Chun et al. 2018). The closest relative of TCPS12 was the strain 40-3 T with a 16S rRNA gene sequence similarity of 99.82%, following by the type strain of *Shewanella baltica* (98.85%). The strain 40-3 T was isolated from seawater in the Arctic and not validly described as type strain of *Shewanella arctica* (Kim et al. 2012; Qoura et al. 2014). TCPS13 isolate showed high sequence similarity (99.73%) to the type strain of *Pseudomonas fragi*. The analysis of both isolates by MALDI-TOF MS corroborated the species-level identification. A phylogenetic tree (Fig. 1) was constructed in order to assess the taxonomic position of each isolate with members most closely related of these genera. Stability analysis by bootstrap resampling showed that gene trees obtained were stable and well defined. Based on MALDI-TOF MS and phylogenetic results, the strain TCPS12 was found to be a member of the genus *Shewanella* and was most closely related to *Shewanella baltica*, while TCPS13 was supported its affiliation within the genus *Pseudomonas*, belonging to *Pseudomonas fragi*.

Strains TCPS12 and TCPS13 have been deposited in the Spanish Type Culture Collection (CECT) as *Shewanella baltica* CECT 30075 and *Pseudomonas fragi* CECT 30069, respectively.

### Antimicrobial activity of supernatants (AS)

The antimicrobial activity of the supernatants AS12 and AS13 was tested against four foodborne pathogens strains. As shown in Table 1, both samples exhibited antimicrobial activity against both Gram-positive and Gram-negative strains, with higher activity of AS13 compared to AS12. The growth of *E. faecalis*, *S. aureus* and *E. coli* was significantly reduced by *P. fragi* supernatant compared to *S. baltica* supernatant. This may be due to the great variability of results obtained with AS12. No significant differences were obtained between AS12 and AS13 activity against *S.* Enteriditis growth. Control solutions at pH 7 and pH 3 showed no significant differences in their inhibitory activity against test strains. These results demonstrated that the acidic pH of these suspensions was not responsible for the antimicrobial activity against the test strains.

**Table 1:** Inhibition of growth of foodborne pathogens by supernatants AS12 and AS13 and pH

Treatment	*E. faecalis*	*S. aureus*	*E. coli*	*S.* Enteritidis	Mean	SD
AS12	46.22	51.08	41.37	68.84	51.88	11.98
AS13	95.61***	97.49**	96.11*	95.29	96.13	0.97
pH 7	6.97	4.20	1.12	2.21	3.63	2.57
pH 3	5.82	3.34	3.92	1.40	3.62	1.82

*SD* standard deviation

^***^Significant difference *p* < 0.001

^**^Significant difference *p* < 0.01

^*^Significant difference *p* < 0.05

The addition of AS12 and AS13 supernatants to a culture of *E. faecalis* at the early exponential growth phase resulted in 1.5 and 2 log cfu/mL reduction, respectively, related to the control (data not shown).

Since the supernatant of *P. fragi* CECT 30069 (AS13) showed much higher antimicrobial activity than the supernatant of *S. baltica* CECT 30075 (AS12) against all tested strains, it was used to evaluate the stability of supernatant antimicrobial activity to environmental conditions and to a partial characterization of the antimicrobial compound.

### Stability of AS13 antimicrobial activity

**Fig. 2:**
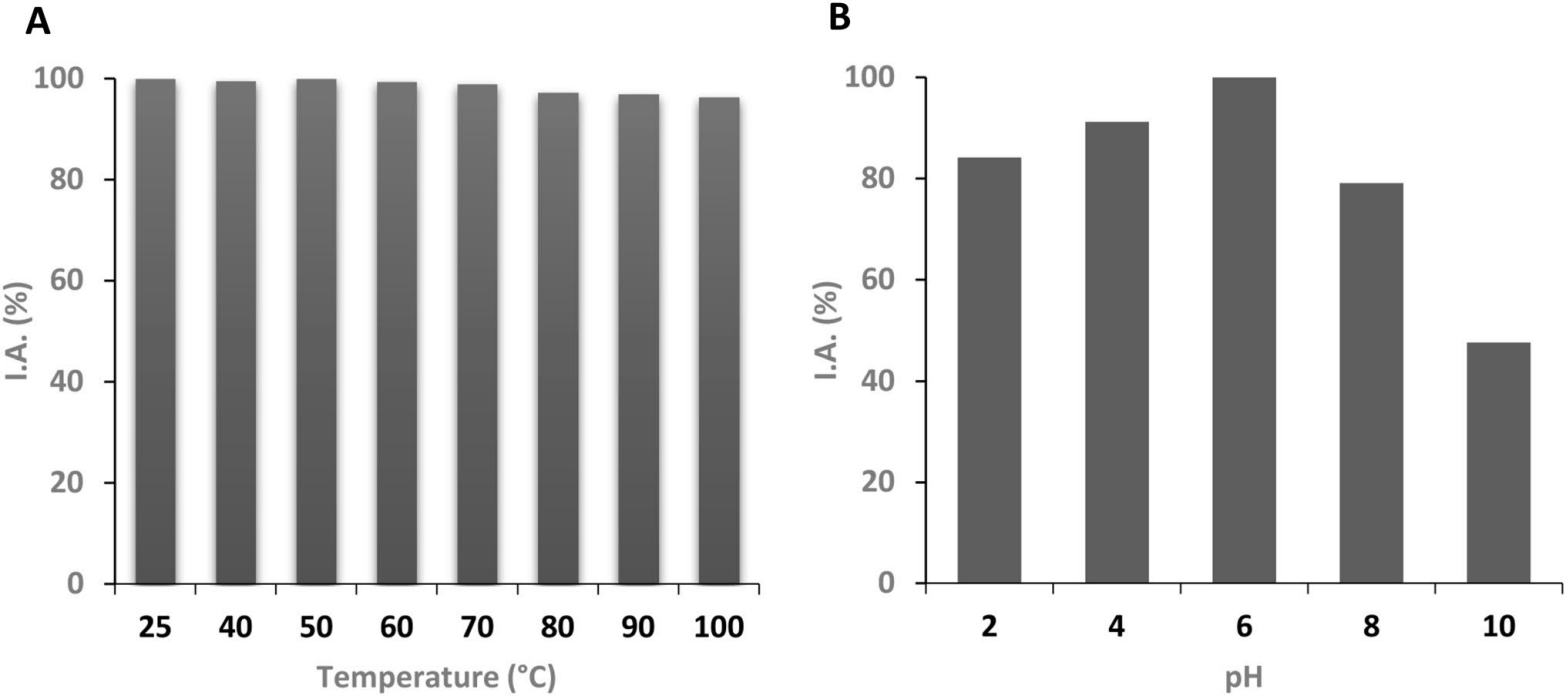
Stability of the AS13 antimicrobial solution to different temperatures (**A**) and pHs (**B**)

The stability of AS13 antimicrobial activity to both different temperature and pH values against *E. faecalis* ATCC 29212 is reported in Fig. 2 and Supplementary Tables S2 and S3. The supernatant AS13 exhibited a very good stability up to the highest tested temperature (100 °C) (Fig. 2A; Table S2). In addition, the solution retained its activity much better in acidic than alkaline pH conditions during a 2-h incubation (Fig. 2B; Table S3). These findings show that the supernatant of *P. fragi* CECT 30069 is a promising candidate for further studies as food preservative, because its antibacterial activity remains stable at different environmental conditions that could be encountered during food processing.

### Effect of enzymes on antimicrobial activity of AS13

The effect of different enzymes on the antimicrobial activity of AS13 against the test strains is shown in Table 2. The inhibition of growth of *E. faecalis* was significantly reduced by α-chymotrypsin and proteinase K to 26.5% and 31.7%, respectively. A loss of about 71% against *E. coli* was observed after treating the supernatant with α-chymotrypsin. Although we observed a decrease in antimicrobial activity of AS13 in the rest of the treatments and strains the results were not significantly different. α-chymotrypsin and proteinase K are serinproteases with hydrolitic activity on peptidic bounds with hydrophobic chains at the terminal carboxyl. These results suggest that the antimicrobial compound could most likely be a glycoprotein. Our results are in agreement with those reported by Chin-A-Woeng et al. (2000): phenazine, an antibiotic produced by different species of *Pseudomonas*, mainly from *P. fragi* group, is a glucosidic compound sensitive to serinproteases activity.

**Table 2:** Percentage of residual AS13 antimicrobial activity after treatment with enzymes

Treatment	*E. faecalis*	*S. aureus*	*E. coli*	*S.* Enteritidis
No one	100 (89.51)	100 (95.2)	100 (90.42)	100 (92.63)
α-Chymotrypsin (2.5 mg mL^−1^)	26.50*	41.56	29.20*	62.12
Lipase (2.5 mg mL^−1^)	60.95	95.38	56.31	77.02
Trypsin (2.5 mg mL^−1^)	68.50	63.91	56.34	88.62
Proteinase K (1 mg mL^−1^)	31.66*	41.37	40.52	60.33
Amylase (2.5 mg mL^−1^)	58.06	54.58	43.31	66.88

Antimicrobial activity of supernatant AS13 without the addition of enzymes is 100%

In brackets: absolute inhibitory activity (I.A.)

^*^Significant difference *p* < 0.05

## Conclusions

In the present study, two novel psychrotrophic bacteria, *Pseudomonas fragi* CECT 30069 and *Shewanella baltica* CECT 30075, were isolated from river trout samples, which produce substances that inhibit Gram-negative and Gram-positive pathogens. The antimicrobial activity of the supernatant of *P. fragi* (AS13) against four food pathogens was higher than the activity of the supernatant of *S. baltica* (AS12). AS13 showed a good both temperature and pH stability, thus this EPS offers promising applications as food preservative.

*Shewanella* species are widely distributed in marine and freshwater environments and they have been described as a normal component of the surface microbiota of fish and as an important fish spoilage agent (Duan et al. 2018; Fonnesbech Vogel et al. 2005; Gonzalez et al 1999; Koziñska and Pekala 2004; Pekala et al. 2015; Skrodenyte-Arbaciauskiene et al. 2006; Ziemke et al. 1998). However, to our knowledge, it is the first time that a psychrotrophic *S. baltica* strain with antibiotic activity has been isolated from fish. More work will be necessary to study the characteristics of its EPS with antibacterial activity and its stability to different environmental conditions. Furthermore, the two new cold-adapted strains described in this work and their enzymes could be very useful tools for the industry.

## Supplementary Information

Below is the link to the electronic supplementary material.

Supplementary file1 (PDF 141 KB)
