# Supplementary material for: Assessing the inhibitory activity of culture supernatants against foodborne pathogens of two psychrotrophic bacteria isolated from river trout
Source: Arch Microbiol. 2022 May 4;204(6):294. doi: 10.1007/s00203-022-02919-5 (PMC9068630; doi:10.1007/s00203-022-02919-5)
Supplement: Supplementary file 1 — Supplementary file1 (PDF 141 KB) [file 203_2022_2919_MOESM1_ESM.pdf]

**Supplementary Table S1** Phenotypic characterization of the two strains isolated from river trout

| Phenotypic characteristic | Test conditions | | TCPS12 | TCPS13 |
|---|---|---|---|---|
| Colony morphology | | | Yellow | White |
| Gram | | | Gram negative rods | Gram negative rods |
| Oxidase | | | + | + |
| Hemolysis | | | γ-hemolysis | γ-hemolysis |
| Motility | 20°C | | + | + |
| | 30°C | | + | + |
| $H_2S$ production | 20°C | | + | - |
| | 30°C | | + | - |
| Nitrate reduction | | | + | - |
| Anaerobic growth at 30°C | | | - | - |
| Aerobic growth | 4°C | TSB | 72h | 72h |
| | | TSA | 72h | 72h |
| | 15°C | TSB | 24h | 24h |
| | | TSA | 24h | 24h |
| | 20°C | TSB | 24h | 24h |
| | | TSA | 24h | 24h |
| | 30°C | TSB | 24h | 24h |
| | | TSA | 24h | 24h |
| | 35°C | TSB | 72h | 72h |
| | | TSA | 72h | 72h |
| | 37°C | TSB | - | - |
| | | TSA | - | - |
| Tolerance to NaCl | 0% | | 24h | 24h |
| | 1% | | 24h | 24h |
| | 3% | | 24h | 24h |
| | 5% | | 24h | 48h |
| | 8% | | 168h | - |
| | 10 - 15% | | - | - |
| Pyocianin production | | | nd | - |
| Pyoverdine production | | | nd | - |

* "+", means growth and test positive
** "-", means no growth or test negative
*** "nd", means not determined

**Supplementary Table S2** Effect of temperature on the stability of AS13 antimicrobial activity

| Temperature (ºC) | I.A. (%) |
|---|---|
| 25 | 100 |
| 40 | 99,5 |
| 50 | 100 |
| 60 | 99,4 |
| 70 | 98,9 |
| 80 | 97,2 |
| 90 | 96,9 |
| 100 | 96,3 |

I.A.: absolute inhibitory activity

**Supplementary Table S3** Effect of pH on the stability of AS13 antimicrobial activity

| pH | I.A. (%) |
|---|---|
| 2 | 84,2 |
| 4 | 91,2 |
| 6 | 100 |
| 8 | 79,1 |
| 10 | 47,6 |

I.A.: absolute inhibitory activity
